# Mild acute stress prevents the memory impairment induced by long-term isoflurane anesthesia

**DOI:** 10.1515/tnsci-2022-0261

**Published:** 2022-12-01

**Authors:** Tiantian Liu, Yutong Dai, Minhui Xu, Ying Chen, Tianjiao Xia, Xin Zhao

**Affiliations:** Department of Anesthesiology, Nanjing Stomatological Hospital, Medical School of Nanjing University, Nanjing, China; Medical School of Nanjing University, Nanjing, China; Department of Anesthesiology, Affiliated Drum Tower Hospital, Medical School of Nanjing University, Nanjing, China

**Keywords:** POCD, acute stress, isoflurane, memory impairment

## Abstract

**Objectives:**

Long-term isoflurane anesthesia exposure could result in postoperative cognitive dysfunction (POCD). Preoperative stress is also reported to be a risk factor of POCD. However, it is unknown whether acute stress could impair memory after long-term isoflurane anesthesia.

**Methods:**

In this study, we categorized the mice with acute stress into mild (30 min restraint stress), moderate (60 min restraint stress), and severe (120 min restraint stress) stress groups and then we used Open-Field Test (OFT) to detect whether different scales of acute restraint stress successfully induced acute stress in mice. The memory performance of mice was measured using contextual and cued memory test, and the brain-derived neurotrophic factor protein levels of hippocampus was detected by Western blot.

**Results:**

We verified that mild stress has pro-cognitive effect, but severe stress has amnestic effect. Moreover, we found that mild and moderate other than severe acute stress could partially attenuate the memory impairment induced by long-term isoflurane anesthesia.

**Conclusion:**

Mild and moderate acute stress could partially attenuate the memory impairment induced by long-term isoflurane anesthesia.

## 1 Introduction

Postoperative cognitive dysfunction (POCD) is characterized by cognitive dysfunction, attention deficit, and executive dysfunction following anesthesia and surgery [1]. Since its first description in 1955 [2], POCD has aroused global concern, due to its association with increased risks of postoperative complications, morbidity, and mortality [3]. According to epidemiological data, up to 26.5% of patients suffer POCD for 1 week after operation, and 9.9% for 3 months [4]. Given the high mortality of POCD [5], there is an urgent need to probe into its pathogenesis. Recently, general anesthesia has been reported to exert detrimental effects on cognition in animal models [6]. Our previous studies have also clarified that long-term isoflurane anesthesia (6 h) could impair spatial memory [7], contextual memory, cued memory, and recognition memory [8].

Accumulating evidence has demonstrated that various etiologies, such as aging, extensive surgery and anesthesia, and low educational level, are involved in the occurrence of POCD [4]. Preoperative stress is also associated with postoperative executive dysfunction and prolonged postoperative recovery [9,10]. Research by Anna et al. shows that more than 40% of cardiac patients experience preoperative stress regarding the disease, surgery, and post-surgery complications [11]. High-level stress, which negatively impacts the central nervous system, is involved in multiple neurological disorders, including depression and posttraumatic stress disorder [12]. By contrast, a lower level of stress seems to be associated with a better cognition in humans and rodents [13–15]. To the best of our knowledge, the effect of acute stress on long-term isoflurane anesthesia-induced POCD has never been explored.

In this study, acute stress was induced by restraint stress in the mouse model, which was divided into mild (30 min restraint stress), moderate (60 min restraint stress), and severe (120 min restraint stress) stress groups. Open-Field Test (OFT) verified the successful induction of acute stress by different scales of acute restraint stress (ARS). Fear conditioning test revealed that mild acute stress exerted a pro-cognitive effect, and mild and moderate acute stress partially attenuated the memory impairment induced by long-term isoflurane anesthesia. Acute stress might modulate the anesthesia-related cognitive disorder via targeting the hippocampal brain-derived neurotrophic factor (BDNF).

## 2 Materials and methods

### 2.1 Animals

In this study, a total of 184 6–7 weeks male C57/BL-6J mice weighing 20–22 g were used. We tried our best to minimize the number of mice. All animals were kept for at least 3 weeks, with a 12 h light/dark cycle (light on at 8:00 and light off at 20:00) in appropriate room temperature (23 ± 1°C) and free access to food and water.

### 2.2 ARS

In this study, we categorized acute stress into three different levels: mild, moderate and severe acute stress induced by 30, 60, and 120 min of restraint stress, respectively. The stressed mice were individually restrained in 50 mL conical, transparent polypropylene tubes that were perforated to provide ventilation. Meanwhile, the corresponding unstressed animals were kept undisturbed in their home cages.

### 2.3 OFT

The OFT was performed as described previously [16]. Briefly, following the restraint stress, each mouse from the stressed and unstressed group was placed in the center of the custom-made Perspex open field with white sides and base (45 cm × 45 cm × 20 cm) for 10 min. An overhead camera recorded the locomotor and exploratory activities of the mice. The time spent in the central zone and the distance traveled in the central zone were automatically recorded with ANYMAZE software. After each trial, the apparatus was cleaned with 75% ethanol solution to eliminate the animal odors and residues.

### 2.4 General anesthesia

Mice were put in plexiglass chambers and anesthetized under a mixture of 1.3% isoflurane (Tocris, 9A/180370) and 100% oxygen (2–3 L/min). The anesthesia procedure lasted for 6 h, during which an anesthesia gas monitor supervised the inspiratory concentration of isoflurane. We also monitored the respiratory activities, an important vital sign, of mice during the whole procedure.

### 2.5 Fear conditioning test

The contextual and cued fear conditioning tests were carried out using Panlab Fear Conditioning System (Harvard Apparatus, Spain). Each mouse was trained and tested only once throughout the fear conditioning experiments to avoid familiarization. During the training phase, each mouse was placed in the conditioning chamber and allowed to freely explore the chamber for 3 min followed with a 30 s pulsating tone (80 dB, 2,000 Hz) which ended with a 2 s mild foot shock (0.75 mA). After that, the mice stayed in the chamber for another 1.5 min. Twenty-four hours later (testing phase), the mice were placed in the former chamber for a total of 5 min without sound and shock for the contextual memory test. Two hours later, mice were placed in a new environment, in which the light changed, and each of the chamber walls was installed with a different colored mental board for the cued memory test. Following 3 min free exploration, the same auditory stimulation (80 dB, 2,000 Hz) was given to the mice for 30 s. Animal’s freezing behavior, defined as an utterly immobile posture except for breathing in the test chamber, was continuously recorded using the Packwin 2.0 software. The freezing behavior was then converted to the percentage of freezing time to assess mice’s learning and memory.

### 2.6 Western blot

After the last behavioral test, all mice were given euthanasia. The hippocampus of mice was isolated and immediately stored at −80°C. Western blot was performed to measure the protein levels of hippocampal BDNF. In brief, the hippocampus tissues were washed to wipe out the circulating blood, dried the surface water, weighed, and homogenized in cold radioimmunoprecipitation assay (RIPA) lysis buffer (Beyotime, China) with proteinase and phosphatase inhibitors (EMD Millipore, USA), and then centrifuged to acquire protein supernatants. The total protein was quantified using the bicinchoninic acid method (Beyotime, China). Equal amounts of protein (40 μg) were loaded into each lane of 10% SDS-PAGE gel and separated by electrophoresis. Proteins were subsequently electrotransferred to a polyvinylidene fluoride membrane (EMD Millipore, USA). The membranes were blocked with 5% (w/v) milk for 1 h, probed by primary antibodies against BDNF (1:1,000, Abcam) and β-actin (1:1,000, Abcam) and HRP-conjugated secondary antibodies diluted in 5% (w/v) milk.


**Ethical approval:** The research related to animals’ use has been complied with all the relevant national regulations and institutional policies for the care and use of animals. Experiments were performed with the approval of the Laboratory Animal Ethics Committee of Drum Tower Hospital, and all experimental procedures were conducted according to the EU Directive 2010/63/EU for animal experiments. The experiment was approved by the Institutional Animal Care and Use Committee, Nanjing University, China (approval No. 20171102).

## 3 Experimental design

### 3.1 Experiment 1

In this experiment, a restraint stress model was applied to induce acute stress in mice. Twenty-four mice were randomly divided into 4 groups (6 mice per group): control group (con), mild stress group (mice subjected to 30 min restraint stress), moderate stress group (mice subjected to 60 min restraint stress), and severe stress group (mice subjected to 120 min restraint stress). To assess the efficacy of the restraint model, the mice were put in the open field immediately after the restraint stress, and an overhead camera recorded their locomotor and exploratory behaviors.

### 3.2 Experiment 2

To verify the effects of different levels of ARS on the fear conditioning memory, 72 mice were randomly divided into the control group, mild stress group, moderate stress group, and severe stress group (18 mice per group). Each group was subjected to different scales of ARS, and then was further split into three subgroups to measure the freezing behavior of mice using the fear conditioning test. One subgroup was measured on the first day after stress exposure (Day 1, *n* = 6), one subgroup on the third day after stress exposure (Day 3, *n* = 6), and another subgroup on the seventh day after stress exposure (Day 7, *n* = 6).

### 3.3 Experiment 3

To verify the effects of different levels of ARS on the anesthesia-related memory deficits, 88 mice were randomly divided into the control group (Con), anesthesia group (Ane), mild stress + Ane group, moderate stress + Ane group, severe stress + Ane group (16–18 mice per group). Stressed mice were subjected to different scales of restraint stress 30 min before anesthesia. The anesthesia mice were exposed to 6 h isoflurane anesthesia. Similar to Experiment 2, each group was further divided into 3 parts to conduct fear conditioning test on Day 1, Day 3, and Day 7 after stress and anesthesia exposure, respectively. After behavioral tests, all mice were given euthanasia, and the hippocampus was isolated and rapidly preserved at −80°C. We then detected the relative protein levels of BDNF in the hippocampus of mice using western blot.

### 3.4 Statistical analyses

All data were expressed as the mean value ± standard deviation (SD). Statistical analyses were conducted by one-way ANOVA followed by Bonferroni *post hoc* tests using SPSS 17.0 software. Significance was defined when *P* < 0.05.

## 4 Results

### 4.1 Effects of different levels of ARS on open-field behavior of mice

**Figure 1:**
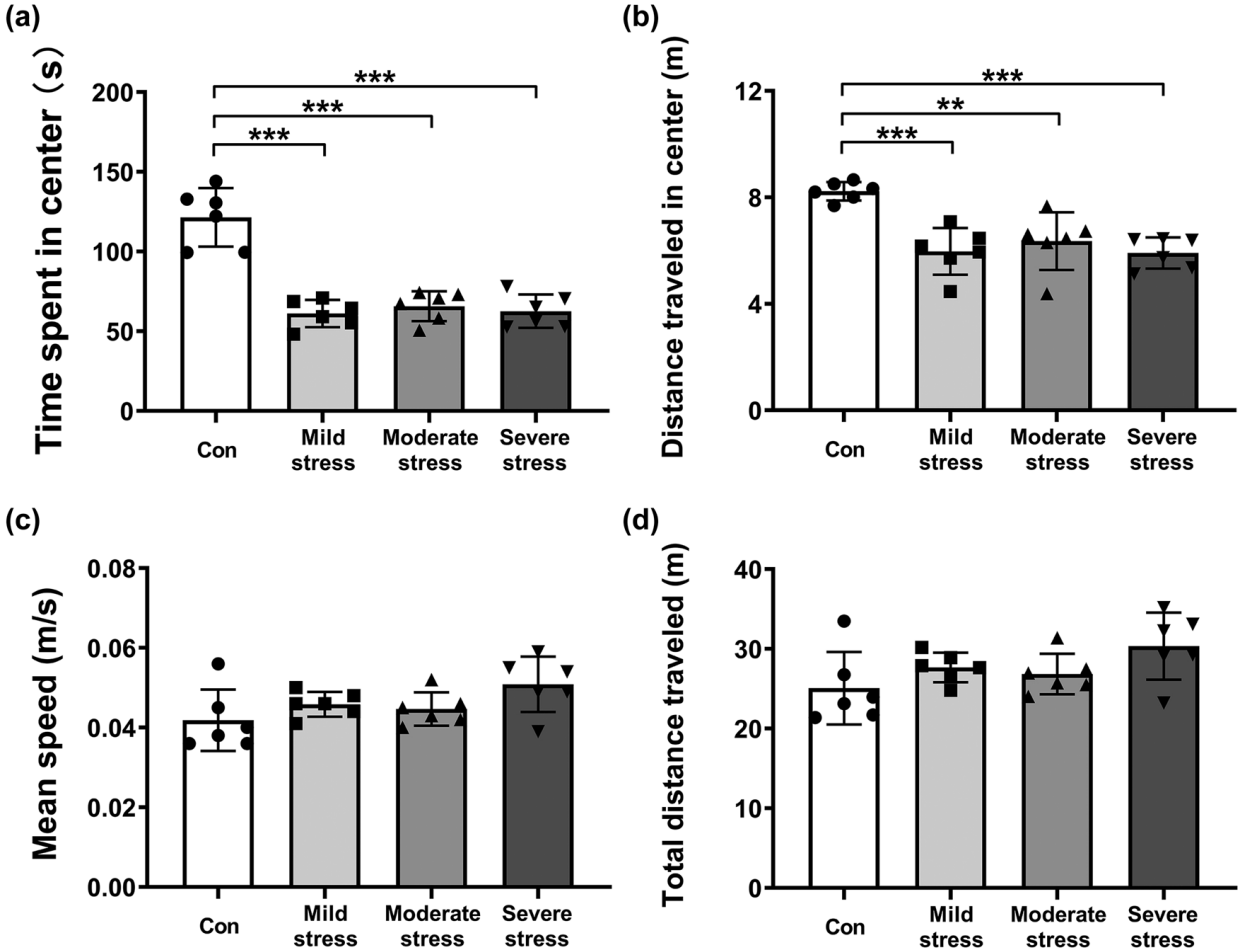
Effects of different levels of acute restraint stress on open-field behavior of mice. The time spent in the center of the open field (a). The distance traveled in the center of the open field (b). The mean speed moved in the open field (c). Total distance traveled in the open field (d). All data are presented as mean value ± SD (*n* = 6/group). ^**^ *P* < 0.01, ^***^ *P* < 0.001, compared with Con group (One-way ANOVA followed by Bonferroni *post hoc* test).

To affirm the efficacy of ARS on mice, we measured the open-field behaviors of mice in the OFT. One-way ANOVA analysis revealed the significant effect of ARS on the time spent in the center zone (*F*_(3,23)_ = 33.744, *P* = 0.000, Figure 1a), and the *post hoc* analysis indicated a decrease in the mild stress group (*P* = 0.000), moderate stress group (*P* = 0.000), and severe stress group (*P* = 0.000) as compared to the Con group, respectively. Similarly, ARS significantly affected the distance moved in the center zone (*F*_(3,23)_ = 11.940, *P* = 0.000, Figure 1b), with a significant decrease in mild stress group (*P* = 0.000), moderate stress group (*P* = 0.003), and severe stress group (*P* = 0.000) in relation to the Con group. No significant difference was seen among all groups in all traveled distances and average speed throughout the OFT (Figure 1c and d).

### 4.2 Effects of different levels of ARS on contextual memory and cued memory of mice

**Figure 2:**
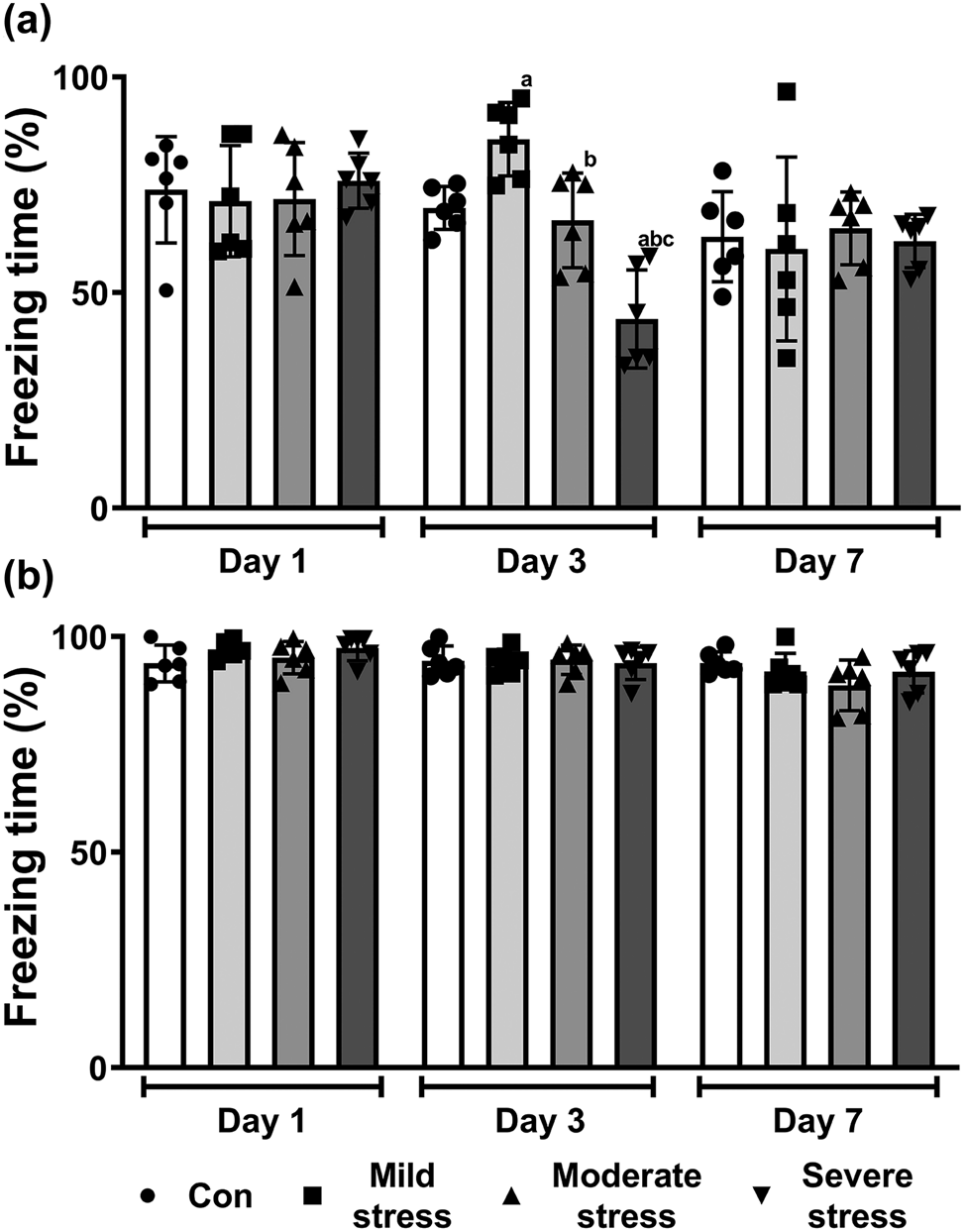
Cognitive performance of mice after induction of different levels of acute restraint stress. Freezing percentage in contextual fear conditioning test (a) and cued fear conditioning test (b). All data are presented as mean value ± SD (*n* = 6/group). ^a^ *P* < 0.05, compared with the Con group; ^b^ *P* < 0.05, compared with the mild stress group; ^c^ *P* < 0.05, compared with the moderate stress group (One-way ANOVA followed by Bonferroni *post hoc* test).

We affirmed the fear conditioning of mice on Day 1, 3, and 7 after different levels of ARS, respectively. In the contextual fear condition test, a significant increase in freezing time was found in the mild stress group (*P* = 0.047), and a significant decrease in freezing time was found in the severe stress group (*P* = 0.001) on Day 3 after anesthesia compared to the Con group (Figure 2a). However, no significant difference in freezing time was found among the 4 groups on Day 1 and Day 7 after anesthesia (*P* > 0.05, Figure 2a). ARS did not impact the freezing behavior of mice in the cued fear conditioning test (*P >* 0.05, Figure 2b).

### 4.3 Effects of different levels of ARS on contextual memory and cued memory of mice after long-term isoflurane anesthesia

**Figure 3:**
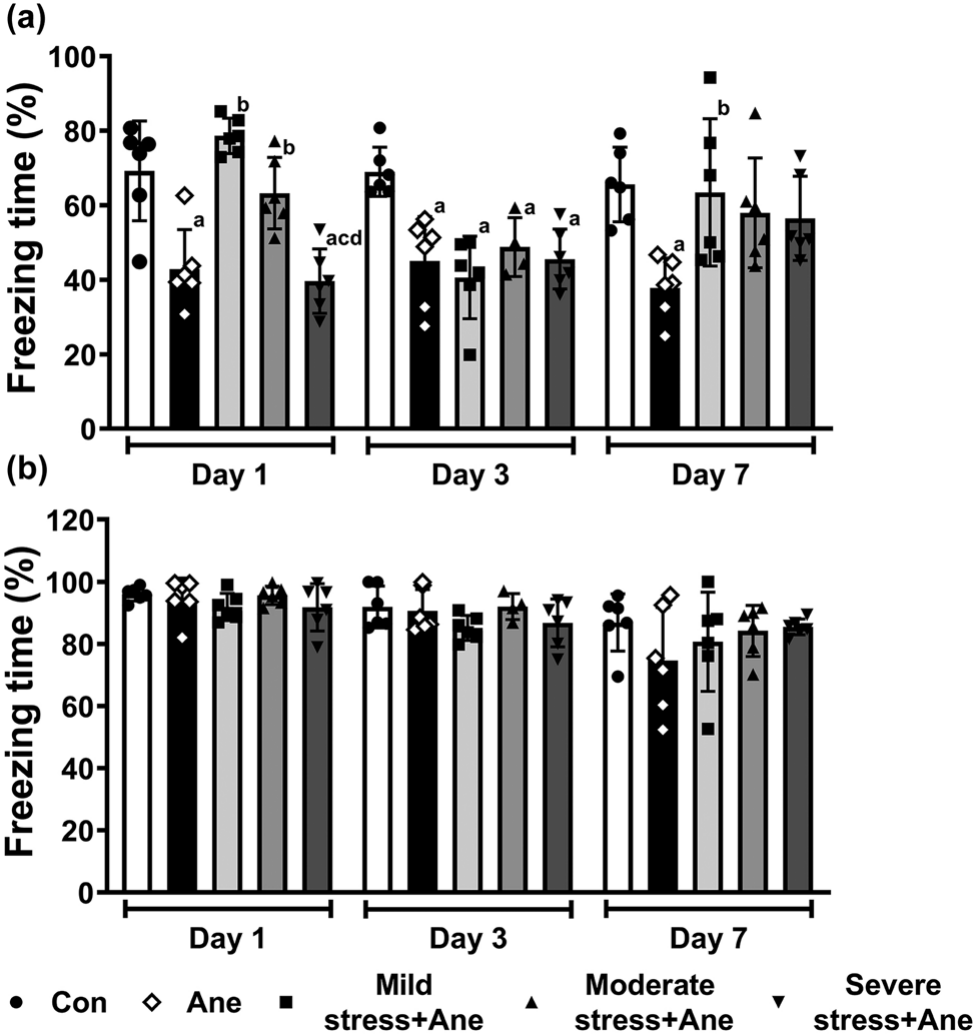
Cognitive performance of mice after induction of different levels of acute restraint stress. Freezing percentage in contextual fear conditioning test (a) and cued fear conditioning test (b). All data are presented as mean value ± SD (*n* = 4–6/group). ^a^ *P* < 0.05, compared with the Con group; ^b^ *P* < 0.05, compared with the Ane group; ^c^ *P* < 0.05, compared with the mild stress + Ane group; ^d^ *P* < 0.05, compared with the moderate stress + Ane group (One-way ANOVA followed by Bonferroni *post hoc* test).

Furthermore, to investigate whether acute stress can modulate the anesthesia-related memory impairment, mice were subjected to different levels of ARS following long-term isoflurane anesthesia, and their memory performance was tested in fear conditioning test on Day 1, Day 3, and Day 7 after anesthesia, respectively (Figure 3). In the contextual fear conditioning test, the Ane group exhibited a significant reduction in freezing time compared with the Con group on Day 1 (*P* = 0.001), Day 3 (*P* = 0.002), and Day 7 (*P* = 0.014) after anesthesia, which was consistent with our previous studies [8,17] (Figure 3a). Moreover, a significant increase in freezing time was presented in mild stress + Ane group (*P* = 0.000) and moderate stress + Ane group (*P* = 0.014) compared with the Ane group on Day 1 after anesthesia (Figure 3a). A significant increase in freezing time was also seen between the mild stress + Ane group and the Ane group on Day 7 after anesthesia (*P* = 0.028, Figure 3a). However, on Day 3 after anesthesia, all levels of restraint stress exposure before anesthesia did not exhibit a significant difference in freezing time compared to those of mice with anesthesia exposure only (*P* > 0.05, Figure 3a). Prior-anesthesia exposure to ARS did not impact the freezing behavior of aestheticized mice in the cued fear conditioning test (*P* > 0.05, Figure 3b).

### 4.4 Effects of ARS and anesthesia on protein levels of BDNF in the hippocampus of mice

**Figure 4:**
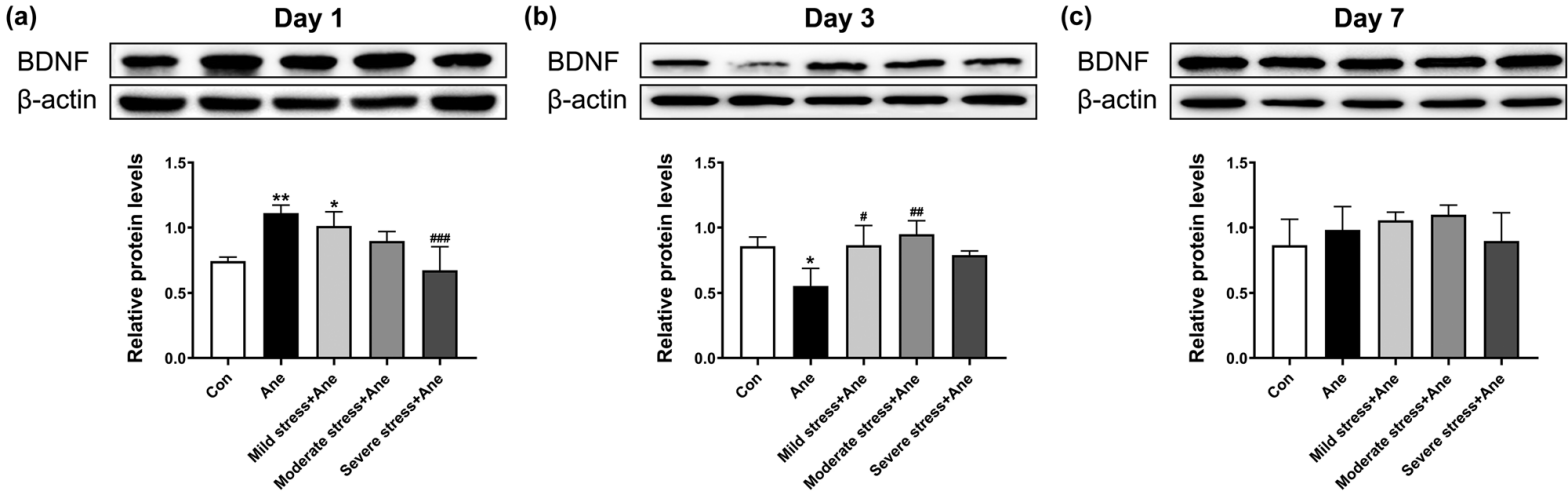
Effects of acute restraint stress and anesthesia on BDNF protein levels in the hippocampus of mice. Relative protein levels of BDNF in the hippocampus of mice detected on Day 1 (a), Day 3 (b), and Day 7 (c) after anesthesia. All data are presented as mean value ± SD (*n* = 4/group). *P < 0.05, **P < 0.01, compared with the Con group; ^#^ *P* < 0.05, ^##^ *P* < 0.01, ^###^ *P* < 0.001, compared with the Ane group (One-way ANOVA followed by Bonferroni *post hoc* test).

We further determined the BDNF protein levels in the hippocampus of mice using western blot. On Day 1 after anesthesia (Figure 4a), a significant increase in BDNF protein level was found in Ane group (*P* = 0.002) and mild stress + Ane group (*P* = 0.023) compared with Con group, and severe stress + Ane group exhibited a significant decrease in BDNF protein level (*P* = 0.000) compared with Ane group. On Day 3 after anesthesia (Figure 4b), Ane group presented a significant decrease in BDNF protein level compared with Con group (*P* = 0.012), and a significant increase in BDNF protein level was displayed in mild stress + Ane (*P* = 0.010) and moderate stress + Ane group (*P* = 0.001) compared with Ane group. On Day 7 after anesthesia, no significant difference was seen among groups (P > 0.05, Figure 4c).

## 5 Discussion

Growing evidence suggests that extensive anesthetic exposure brings cytotoxicity to the brain and causes cognitive dysfunction [18,19]. As reported in the previous studies, preoperative stress is associated with increased postoperative complications and prolonged postoperative recovery in clinical practice, especially for surgical patients [9,10]. Although acute stress has been demonstrated to affect learning and memory in both humans and animals [20–22], the impact of different scales of acute stress on memory disorders after long-term anesthetic exposure is still poorly understood. In this study, we found that pre-anesthesia exposure to mild acute stress could protect the mice from contextual memory impairments on Day 1 and Day 7 after long-term isoflurane anesthesia and the stress and anesthesia exposure could change the BDNF protein levels in the hippocampus of mice.

The animal model of restraint stress is commonly employed to induce acute or chronic stress [23]. Impaired exploratory activity in OFT of mice after ARS has been verified [24]. Our study also found that mild-, moderate-, and severe-ARS all significantly decreased the distance moved and the time spent in the center of the field among ARS mice compared with non-stress mice. Meanwhile, similar levels of locomotor activity were found when comparing ARS mice and non-stress mice, indicating that the immobility and decreased activity of ARS mice in the central area could not be attributed to any alteration in locomotor activity. Besides, a previous study demonstrated that ARS could induce a cognitive change in the fear conditioning test [25]. Our current study, noticeably, found that ARS elicited cognitive change on Day 3 after stress induction in a time-dependent manner: mild stress exerted a pro-cognitive effect, moderate stress exerted no effect, and severe stress exerted a pro-amnestic effect on the contextual memory of mice. Our findings were consistent with those of the previous studies, revealing that 30 min exposure to ARS could facilitate the acquisition of paired associate learning [26], and longer exposure to ARS could damage recognition memory (90 min restraint stress) [27] and working spatial memory (120 min restraint stress) [28]. And restraint stress of all three lengths could increase the serum corticosterone levels [28–30]. Previous literature has pointed out that different concentrations of corticosterone were released in rodents (cortisol in humans) after exposure to different levels of ARS and could exert a reversed U-shaped effect on cognitive function [31]. But in this study, the relationship between the corticosterone levels that could also show the levels of perceived stress after different lengths of ARS and the effect of stress and anesthesia on cognition has not been addressed, which is worth studying in the future.

Extensive anesthetic exposure has been reported to have detrimental effects on brain function, especially memory [19]. For instance, the 4 h sevoflurane inhalation can damage the working memory of mice [32], and repeated exposure to propofol for 6 days can impair the cognition of rats [33]. In line with these observations, our present experiments showed that the 6 h isoflurane anesthesia induced hippocampus-dependent contextual memory deficits from Day 1–7 after anesthesia. Meanwhile, prior exposure to mild and moderate acute stress remarkably rescued the hippocampus-dependent contextual memory deficits on Day 1 after anesthesia, and mild acute stress could also rescue the memory deficits on Day 7 after anesthesia. Hippocampus, a critical brain area involved in learning and memory, is particularly sensitive to stress due to the abundant presence of mineralocorticoid receptors and glucocorticoid receptors (GRs), two classes of corticosteroid receptors that can bind to corticosteroid released after stress exposure [34,35]. A previous study demonstrated that brief restraint stress (30 min) could facilitate long-term potentiation (LTP) in the mouse hippocampus by modulating GRs and ion channels [36], which may explain the anti-amnestic effect of mild acute stress in our study. Besides, animal studies showed that acute stress could alter brain structure and function via multiple interacting mediators, including BDNF [37], tPA [38], CRF [39], and lipocalin-2 [40].

In this study, we focused on the effects of long-term isoflurane anesthesia and different scales of acute stress on BDNF expression in the mouse hippocampus. BDNF, widely expressed in the brain, including the hippocampus plays a critical role in learning and memory [41,42,43]. Previous research has implicated the involvement of BDNF in stress-related mental disorders [44]. In the current study, we found that the hippocampal BDNF expression changed dynamically after the 6 h exposure to isoflurane anesthesia: increasing on Day 1, decreasing on Day 3, and recovering to the control level on Day 7 after anesthesia. Furthermore, the pre-anesthesia exposure to mild acute stress up-regulated BDNF expression on both Day 1 and Day 3 after stress, suggesting a potential mechanism underlying the protective effect of pre-existing mild stress on anesthesia-induced memory deficit.

In conclusion, ARS exposure exerts a time-dependent effect on the contextual memory of mice on Day 3 after stress exposure: mild acute stress improving memory, moderate acute stress having no effect on memory, and severe acute stress damaging memory of mice. Prior exposure to mild and moderate acute stress attenuates the long-term anesthesia-induced memory deficit on Day 1 following anesthesia. Mild acute stress also exerts an anti-amnestic effect on Day 7 following anesthesia. Besides, BDNF in the hippocampus may play a critical role in this process. Our study still has some limitations. For instance, only fear conditioning test was conducted in the study, which is relatively unitary cognitive assessment and could not exclude the affective effect, another hippocampus-based learning test such as T-maze and water maze tests could also be conducted. Meanwhile, given previous research works showed conflicting results related to the effects of stress on female mice in fear conditioning test [45,46], we just used male mice in this study. Sex difference in the effect of acute stress and anesthesia on cognition could be investigated in the future work using more behavior tests.
